# Novel strategy for hepatocyte transplantation using resected organ with hepatocellular carcinoma or cholangiocarcinoma after hepatectomy

**DOI:** 10.20407/fmj.2019-009

**Published:** 2019-11-02

**Authors:** Toki Kawai, Masahiro Ito, Chihiro Hayashi, Naoki Yamamoto, Yukio Asano, Satoshi Arakawa, Akihiko Horiguchi

**Affiliations:** 1 Department of Gastroenterological Surgery, Fujita Health University Bantane Hospital, Nagoya, Aichi, Japan; 2 Fujita Health University, Institute for Comprehensive Medical Science, Toyoake, Aichi, Japan

**Keywords:** Hepatocyte transplantation, Large hepatectomy, Cholangiocarcinoma

## Abstract

**Objectives:**

Although large hepatectomy (i.e., resection of 2–3 segments) is an increasingly common treatment for hepatocellular carcinoma and cholangiocarcinoma, it can lead to liver failure. However, a resected liver may contain large quantities of both normal hepatocytes (NHs) and carcinoma cells. We investigated separating these cell types so that NHs could be used as transplantable cells.

**Materials and methods:**

Cancer cells were developed by immortalizing rat hepatocytes, using an artificial chromosome vector. Cancer cells and primary hepatocytes (PHs) were mixed in a 1:1 ratio, then separated into two groups using fluorescence activated cell sorting (FACS). Normal hepatocytes after FACS (NHaF) and cancer cells after FACS (CAaF) were transplanted into two spots on opposite sides of the backs of nude mice; and also into the spleens of three groups (NHaF, CAaF and controls) of non-albumin rats (NARs), from which we measured blood albumin levels, using ELISA.

**Result:**

The PH and cancer cells were successfully separated using FACS. After separation, cancer cells transplanted subcutaneously in nude mice formed tumors, whereas transplanted PH cells in NARs only produced higher albumin levels.

**Conclusion:**

Transplanted NHaF cells did not produce tumors. However, this cells function was not enough in power for transplant source by this method. Nevertheless, we believe this technique can be improved and used to treat patients successfully.

## Introduction

Large hepatectomy (i.e., resection of 2–3 segments) is increasingly used to treat hepatocellular carcinoma (HCC) and cholangiocarcinoma (CCC). Although the treatment strategy for HCC has shifted from major hepatectomy (i.e., resection of ≥4 segments) to minimum adequate anatomical resection, depending on tumor condition and liver function, some patients still need major hepatectomies. Use of large hepatectomy is challenging, and survival among patients with advanced disease is limited, as patients may experience liver failure.^1,2^

Liver transplantation is the accepted treatment for patients with acute liver failure and liver-based metabolic disorders. However, a shortage of donor organs and the lifelong requirement for immunosuppression are major limitations to liver transplantation. In addition, as loss of native liver removes the possibility of treating it with future gene therapies, alternative therapeutic strategies are needed. A potential alternative to liver transplantation is allogeneic hepatocyte transplantation.^3–8^ The present study focuses on such a purpose for normal hepatocytes (NHs) found in resected liver specimens.

Whereas previous studies have aimed at using tumor antibodies to cure cancer, the present study used tumor antibodies to separate NHs and carcinoma cells, using fluorescence activated cell sorting (FACS); the separated cells were then used for transplantation. Syngeneic transplantation may be possible using hepatocyte transplants to treat liver failure without the use of immunosuppressive drugs. Immortalized cells were used as cancer cells in this study.

## Materials and Methods

### Isolation of primary Lewis rat hepatocytes

Hepatocytes were isolated from Lewis (LEW) rats with an *in situ* two-step perfusion technique previously described by Berry and Friend^9^ and modified by Seglen,^10^ using collagenase (type IV, Sigma Chemical Co., St. Louis). Cell viability was consistently between 85% and 95%, as determined by Trypan blue exclusion.

### Preparation of cancer cells

**Figure 1:**
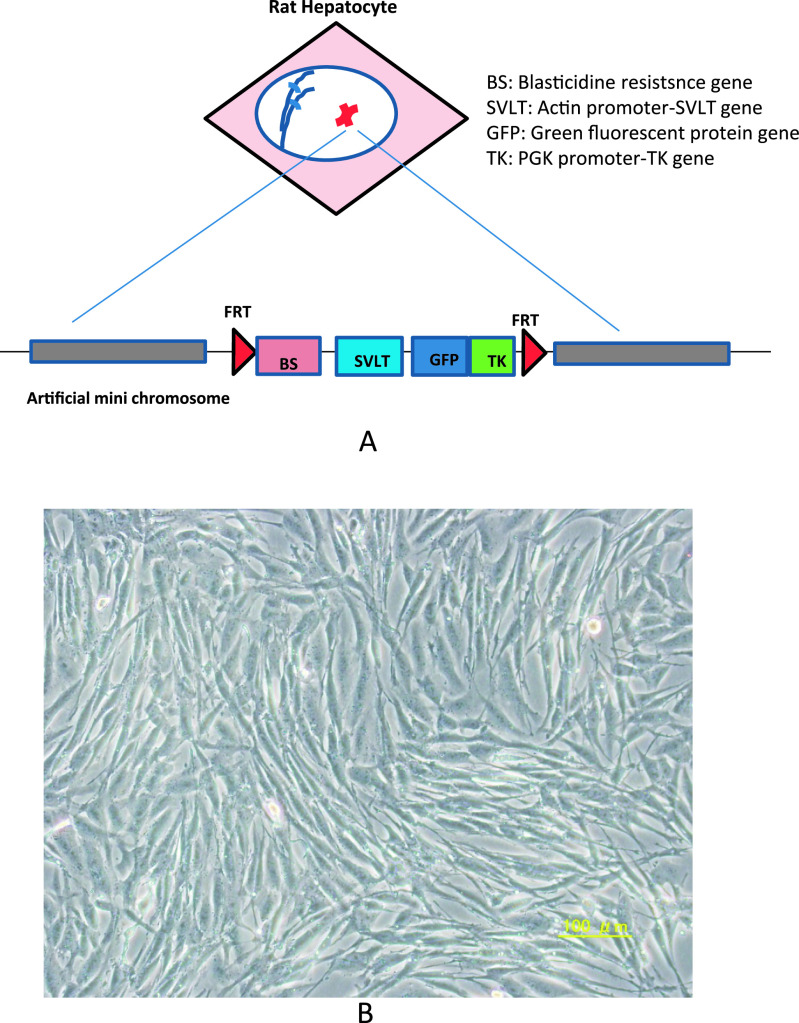
(A) Construction of the human artificial mini chromosome (HAC) that contained the *SV40* T antigen gene in CHO cells. GPF was introduced into immortalized hepatocytes, which were considered cancer cells (CA). (B) Morphological appearance of cultured immortalized cells using a microscope. On Day 3, the immortalized hepatocytes morphologically appeared to be cancer cells.

Immortalized cells was used as cancer cells (CA) in this study. A human artificial chromosome (HAC) was constructed using a bottom-up strategy based on transfecting cloned centromeric alphoid DNA precursors into the HT1080 human cell line.^11^ To provide a platform for gene insertion into the HAC, a HAC that contained Cre/*lox* recombination sites was constructed using cotransfection of alphoid precursors with Cre/*lox* recombination cassettes.^12^ A Flp/FRT cassette was then inserted into one of Cre/*lox* sites on the HAC, resulting in a single Flp/FRT insertion site on the HAC. The HAC containing the gene insertion site was transferred from HT1080 to Chinese hamster ovary (CHO) cells by whole-cell fusion and microcell-mediated chromosome transfer. Next, beta-actin-*SVLT* and *PGK-TK* genes were inserted in the FRT site on the HAC, using blasticidine resistance by transient expression of Flp recombinase in HAC^+^ CHO cells (Figure 1A).

The HAC^+^ CHO cells were grown to 80% confluence; colcemid was then added (0.05 mg/mL). Cells were cultured for 48 h, harvested, and resuspended in serum-free Dulbecco’s Modified Eagle Medium (DMEM) containing cytochalasin-B (20 mg/mL). The suspension was incubated for 5 min at 37°C, after which equal volumes of Percoll (Amersham Biosciences) were added. The suspension was centrifuged at 15,000 rpm for 90 min at 37°C. Microcells were collected by centrifugation at 2000 rpm for 5 min, then resuspended in serum-free DMEM and collected by centrifugation. Microcells were suspended with rat hepatocyte cells in serum-free DMEM and centrifuged at 1500 rpm for 5 min. The pellet was suspended in 1 mL of 30% PEG1500 (Roche Applied Science) and incubated at room temperature for 90 s. We then added 4 mL of serum-free D-MEM and the mixture was centrifuged at 1000 rpm for 5 min. Fused cells were washed twice in serum-free DMEM and plated. After 48 h, the medium was changed to DMEM supplemented with 2 mg/mL blasticidin-S for selection of HAC^+^ cells. The actin promoter-*SVLT* gene and *PGK* promoter-*TK* genes that included the green fluorescent protein (*GFP*) gene were inserted into the FRT site on the HAC, using blasticidine resistance as provided by the transient expression of Flp recombinase in HAC^+^ LEW cells. These cells were regarded as cancer cells. Fluorescence *in situ* hybridization (FISH) was performed according to conventional procedures.^13,14^ Biotin-labeled a21-I alphoid DNA (11-4) and digoxigenin-labeled pBelo-BAC were used as probes to detect HACs.

The morphological appearance of immortalized cells was observed under culture conditions using a microscope. Immortalized hepatocytes morphologically appeared to be cancer cells compared with NHs on Day 3 (Figure 1B).

### Cell separation by FACS

Immortalized HAC^+^ LEW cells (cancer cells) were fluorescently labeled with GFP; therefore, GFP^+^ cells were sorted from unlabeled cells using a FACS (FACS Vantage SE, BD Biosciences, San Jose, CA). Cancer cells (1×10^6^ hepatocytes with SV40 large T antigen+GFP) were then mixed with PHs (1×10^6^) and sorted again using a FACS that detected GFP.

### Transplantation of separated cells into nude mice

Cancer cells separated by FACS (CAaF) and NHs separated by FACS (NHaF) were subcutaneously transplanted in three nude mice, on the right side of the back for CAaF (1×10^4^ in 1 mL DMEM), and on the left side for NHaF (1×10^4^ in 1 mL DMEM). Tumor formation was assessed in all injection sites for three months after transplantation.

### Assessment of cell function by transplantation

A mixture of 1×10^5^ hepatocytes and 1 mL of DMEM were directly injected into spleens of non-albumin rats (NARs: 150–250 g) using 24-gauge needles, to assess whether hepatocyte transplantation could rescue rats from hypoalbuminemia. NARs were divided into three groups and treated as G1: intrasplenic transplantation of 1×10^5^ NHaF (*n*=4); G2: intrasplenic transplantation of 1×10^5^ PH (*n*=4; although it was difficult to ensure the same number of cells per rat); and G3: intrasplenic injection of 0.5 mL of DMEM (*n*=4). The NARs were anesthetized by inhalation of isoflurane (Abbott), and their spleens were exposed via left flank incision. A total of 1×10^5^ hepatocytes were suspended in 1 mL of DMEM (Sigma), then injected into the inferior pole of the spleen using a 24-gauge needle (Terumo) connected to a tuberculin syringe (Terumo). The injection site was ligated with a 3.0 silk suture (Alfresa Pharma) to prevent cell leakage and bleeding. A sham operation involving intrasplenic injection of 1 mL of DMEM and clamping of the splenic vein and artery for 5 min was performed in another group of NARs as a control. Animals that received transplanted hepatocytes or DMEM received intramuscular injections of 1 mg FK506/kg (Tacrolimus; Astellas Pharma, Tokyo, Japan) on alternative days to suppress immune rejection of the allogeneic transplanted hepatocytes.^15^ Blood samples were collected on Days 0, 3, 7, and 14; blood albumin levels were measured using ELISA.

### Animals and chemicals

Inbred male LEW rats (150–250 g), NARs (100–250 g), and nude mice (50–100 g) were obtained from Chubu Kagagu Shizai, Nagoya, Japan, and maintained in the Animal Resource Facility of the Fujita Health University. Animals were maintained on standard laboratory chow on a 12-h light/12-h dark cycle. All procedures performed on the rats were approved by the Fujita Health University Institutional Animal Care and Use Committee and were within the guidelines for the humane care of laboratory animals.

### Statistical analysis

Values are expressed as mean±standard error. Statistical differences were determined as described in the text. Life-table analysis was performed using non-parametric tests (Mann-Whitney *U* Test). *P*<0.05 was considered significant.

## Results

### Cell separation by FACS

**Figure 2:**
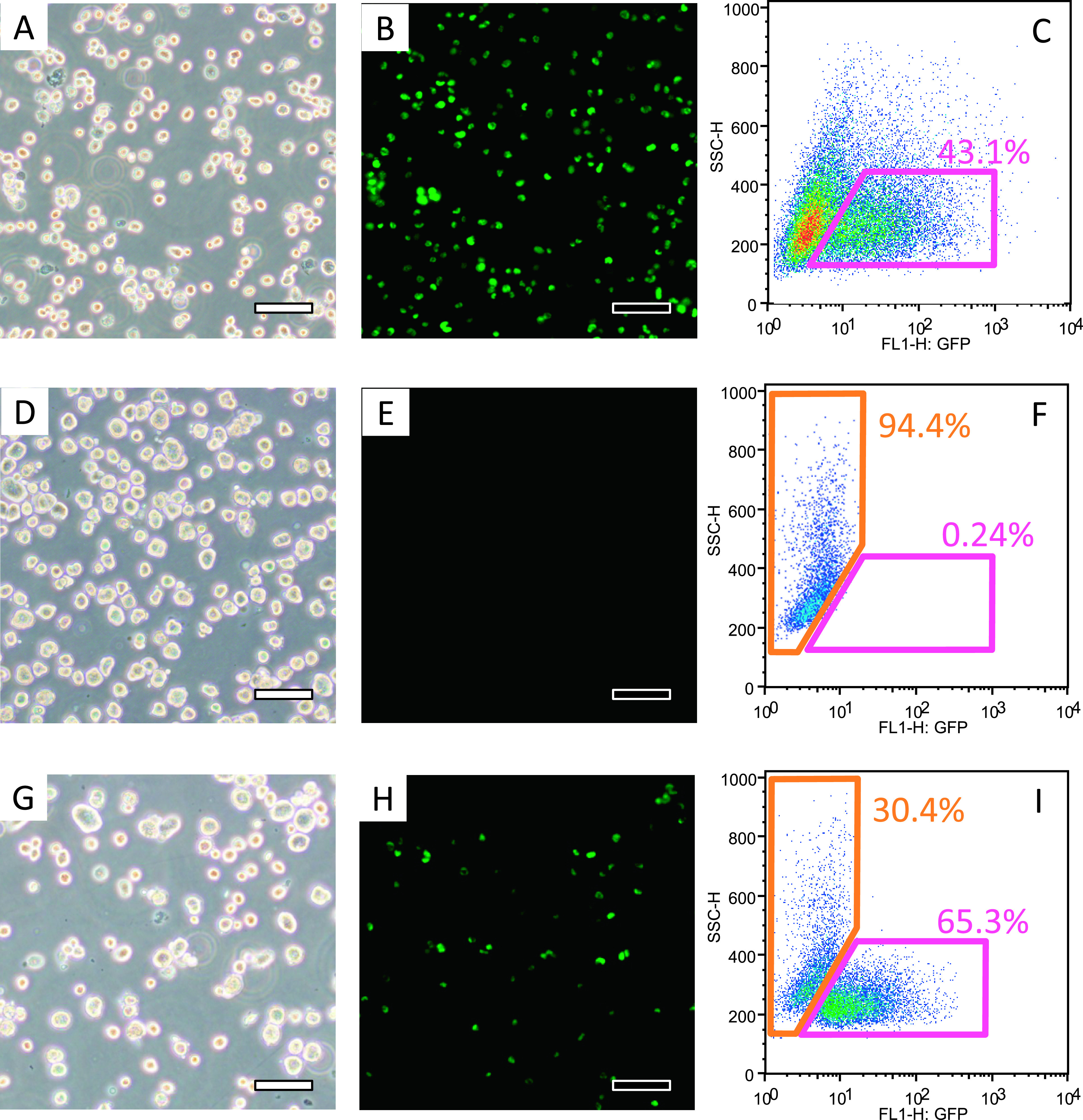
Inverted (A) and fluorescent (B) micrographs showing HAC-containing cells. Flow cytometry analysis showed 43.1% of cells were GFP^+^ cells that contained HAC (C). GFP^+^ cancer cells were selected by FACS. Inverted (D) and fluorescent (E) micrographs showing normal. Primary hepatocytes (PHs) accounted for 94.4% of cells detected in the GFP^–^ region (F). Inverted (G) and fluorescent (H) micrographs of mixed cells show GFP^+^ cancer cells and PHs. Recovery of GFP^+^ cancer cells was 65.3% from the mixed cells, compared with only 30.4% of PHs (I).

FACS was used to prepare three groups for analysis: cancer cell group, PH group, and the experiment group (cancer cells and PH). Cancer cells containing HAC were sorted using FACS to detect GFP. Recovery of GFP^+^ cells (cancer cells) by FACS was 43.1%. Analysis of PHs showed 94.4% GFP^–^ cells, with 92% cell viability. When GFP^+^ cancer cells and PH were mixed (1:1 ratio, 1.0×10^5^ cells/mL each), and GFP^+^ cancer cells were sorted again, the recovery of GFP^+^ cells was 65.3% with a cell viability of 82%, compared with 30.4% of recovered PHs, with a cell survival rate of 72% (Figure 2).

### Assessment of cell function by transplantation

**Figure 3:**
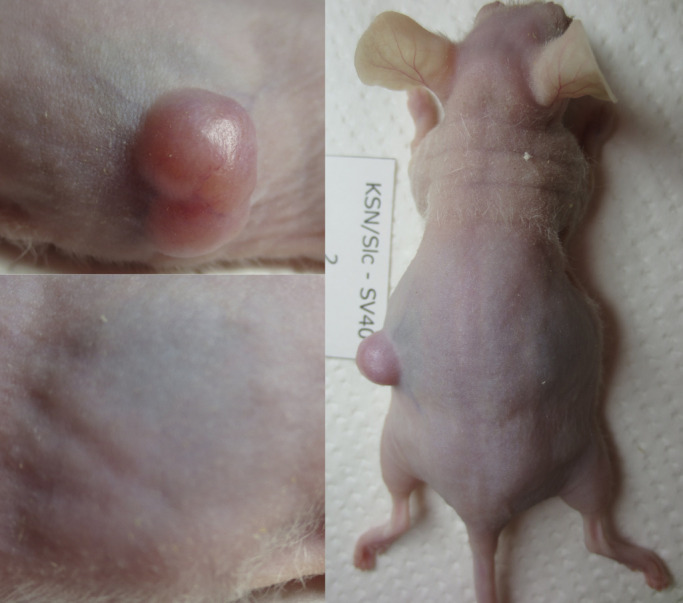
Subcutaneously transplanted normal hepatocytes after FACS (NHaF) in nude mice did not form tumors within three months (left side of back), but subcutaneously transplanted cancer cells after FACS (CAaF) formed tumors (right side).

Over 3 months, subcutaneously transplanted CAaF in nude mice formed tumors and NHaF did not form tumors (Figure 3).

### Purification of cells functioning by transplantation:

**Figure 4:**
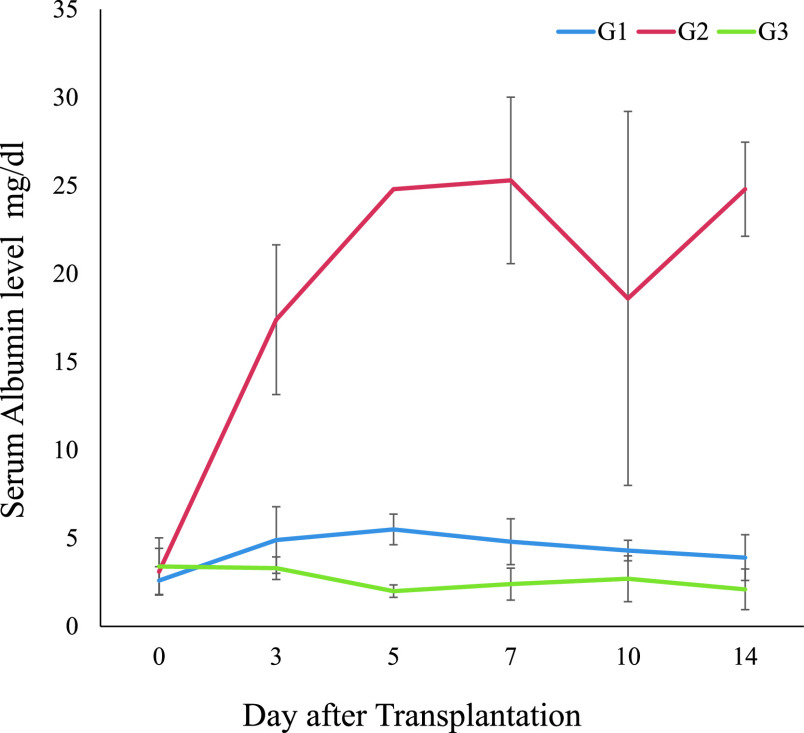
Serum albumin levels: Purification of cells functioning by transplantation. G1 group: without cancer cells using FACS (NHaF; *n*=4; 1×10^5^ cells); G2 group: primary hepatocytes (PHs; *n*=4; 1×10^5^ cells); G3 group, sham (control) operation (*n*=4; DMEM). The G2 group recieved intrasplenic transplantations of 1×10^5^ PH into NARs. Serum albumin levels did not significantly differ between the groups with and without transplantation initially; but significantly differed between NHaF (G1: 4.9±1.8 mg/dL; G2: 17.4±4.2 mg/dL; G3: 3.3±0.6 mg/dL) at one week after transplantation or sham operation (*P*<0.001).

At one week after transplantation or sham operation, serum albumin levels were significantly different between G2 and G3, but not between G1 and G3 (G1: 4.9±1.8 mg/dL, G2: 17.4±4.2 mg/dL, G3: 3.3±0.6 mg/dL; *P*<0.001; Figure 4).

## Discussion

Many studies have suggested that hepatocyte transplantation could serve as an alternative to organ transplantation for patients with liver disease. The success of experimental hepatocyte transplantation has led to several attempts to use hepatocyte transplantation in clinical practice to treat a variety of hepatic diseases (3–5,11,12). However, evidence of transplanted human hepatocyte function has been obtained in only one patient with Crigler–Najjar syndrome type 1, and even then, the amount of bilirubin-UGT enzyme activity derived from the transplanted cells was not sufficient to eliminate the patient’s eventual need for organ transplantation.^16^ In addition, donor livers available for human hepatocyte isolation are limited due to their demand in whole-organ transplantation. One alternative source of transplantable hepatocytes is cells derived from an immortalized hepatocyte cell line or induced pluripotent stem cells.^15,17,18^ Such cells could potentially provide an unlimited supply of well-characterized, pathogen-free liver cells.

The present study investigated the potential use of hepatocyte transplantation for clinical practice. While treatment of cancer patients using the hepatectomy technique is improving, some patients still experience liver failure due to excessive liver resection.

We have shown that PHs and cancer cells are separable by FACS. However, whether sufficiently large quantities of implantable cells can be extracted from small liver samples in clinical practice is unclear. Cell viability and yield after FACS were 72% and 1.5×10^4^ cells (pre-FACS: 92% and 1×10^5^) for PHs; and 82% and 3.2×10^4^ cells (pre-FACS: 100% and 1×10^5^) for cancer cell. Both cell types were damaged through this method (Table 1). NHaF were functionally degraded and could not produce albumin.

In our nude mouse experiment, cancer cells after FACS separation did not form tumors. However, for translational applications, this method alone may not secure enough safety in clinical specific conditions. Further investigations for precise separation techniques are also needed. And also this experiment used immortalized simultaneously cell marked GFP. Labelling and separating 100% of HCC cells is a critical consideration for clinical use. However, for CCC, our current labelling method may be feasible for clinical use. We anticipate that this technique will be improved and eventually used to successfully treat patients, save lives and cure cancer.^19–21^

## Figures and Tables

**Table1:** Cell viability and yield were 92% and 1×10^5^ cells before FACS, and 72% and 1.5×10^4^ cells afterwards for primary hepatocytes (PHs); and 100% and 1×10^5^ cells before, and 82% and 3.2×10^4^ cells afterwards for cancer cells.

	Primely Hep.	Cancer Cell
Pre FACS Viability (%)	92%	100%
Yeild	1×10^5^ cell	1×10^5^ cell
Post FACS Viability (%)	72%	82%
Yeild	1.5×10^4^ cell	3.2×10^4^ cell
